# Highly Efficient Enrichment of Heterocyclic Aromatic Amines in Meat Products Using the Magnetic Metal—Organic Framework Fe_3_O_4_@MOF-545-AMSA

**DOI:** 10.3390/molecules30081705

**Published:** 2025-04-10

**Authors:** Yang Wang, Ying Liu, Ziyan Chen, Shan Liang

**Affiliations:** Beijing Engineering and Technology Research Center of Food Additives, School of Food and Health, Beijing Technology and Business University (BTBU), Beijing 100048, China; wy777800@163.com (Y.W.); lyly0817@126.com (Y.L.); ziyanc2023@163.com (Z.C.)

**Keywords:** magnetic nanoparticles, Fe_3_O_4_@MOF-545-AMSA, meat products, UPLC-MS/MS, heterocyclic aromatic amines, high-throughput testing

## Abstract

Heterocyclic aromatic amines (HAAs), known for their mutagenic and carcinogenic potential, are formed during the heating of protein-rich food items. Detecting HAAs swiftly and accurately poses challenges due to complex food matrices and low HAA concentrations. In this study, a simple and efficient magnetic solid-phase extraction (MSPE) strategy was developed for the simultaneous isolation and enrichment of three HAAs such as 2-amino-3,4,8-trimethylimidazo [4,5-f]quinoxaline (4,8-DiMeIQx), 2-amino-3,8-dimethylimidazo [4,5-f]quinoxaline (MeIQx), and 2-amino-3-methylimidazo [4,5-f]quinoline (IQ) in processed meats, employing the magnetic covalent organic framework Fe_3_O_4_@MOF-545-AMSA as an adsorbent. It was synthesized via a solvothermal method, with Fe_3_O_4_ as the magnetic core. Its building blocks are as follows: zirconium (Zr) as the coordination metal ion, tetrakis(4-carboxyphenyl)porphyrin and benzoic acid as organic ligands, and aminomethanesulfonic acid (AMSA). This composite captures targeted HAAs efficiently by exploiting the unique porous MOF-545-AMSA structure, specific metal–ligand coordination, and AMSA’s amino and sulfonic acid groups. The quantification of HAAs was achieved through the combination of Ultra-Performance Liquid Chromatography–Tandem Mass Spectrometry (UPLC-MS/MS) and MSPE, demonstrating satisfactory linearity (R^2^ ≥ 0.9917), high recovery rates (83.7–111.0%), and low detection limits (0.1–1.0 μg/kg). Moreover, an automated high-throughput detection system was developed using MSPE to assess the presence of HAAs in meat products.

## 1. Introduction

Heterocyclic aromatic amines (HAAs) are a group of heterocyclic compounds which contain aromatic amino groups formed from carbohydrates and amino acids during high-temperature cooking [1]. They are commonly present in protein-rich, heat-treated meat products. Besides poultry and fish, red meat like beef, pork, and lamb can also contain HAAs [2,3]. More than 30 HAAs have been identified so far [4], with some, such as PhIP, MeIQ, and MeIQx, classified as potentially carcinogenic (Group 2B) by the International Agency for Research on Cancer (IARC), and the IARC classifies IQ as probably carcinogenic (Group 2A) [2]. Studies have shown that certain subclasses of HAAs exhibit specific neurotoxic effects [5,6]; for example, long-term exposure to PhIP at dosages exceeding 5 μg/kg body weight may induce Parkinson’s disease [7] and Alzheimer’s disease [8]. Consequently, there is a critical need for reliable, cost-effective methods to rapidly detect HAAs in meat products.

The low concentration of HAAs in food and their susceptibility to interference from complex food matrices pose significant challenges for extraction and accurate detection [1]. Effective pretreatment methods are essential to reduce impurities and improve assay sensitivity. The current techniques for isolating HAAs in meat products include liquid–liquid extraction (LLE) [9], solid-phase extraction (SPE) [10] and microwave-assisted extraction [11]. Among these, SPE is utilized due to its effective purification capabilities. However, despite its efficient enrichment properties, SPE is time-intensive and involves costly absorbents. Thus, further research is necessary to develop a cost-effective, user-friendly and automated method for HAA extraction.

Innovations in extraction methods are heavily reliant on advancements in adsorbent technology, with nanoparticles (NPs) presenting a diverse array of absorbent options. NPs have gained attention in the food industry due to their significant specific surface area and ultra-small size [12]. Metal–organic frameworks (MOFs) are crystalline materials with adjustable pore sizes, formed through coordination between central metal ions and organic ligands [13]. MOFs display higher porosity, stronger adsorption, and better reuse performance stability than other nanomaterials, such as zeolites, silica-based materials, and carbon-based materials [14]. Zhou et al. (2006) pioneered the synthesis of Cu(4-C_5_H_4_N-COO)_2_H_2_O)_4_ using an MOF material as an SPE absorbent for the efficient enrichment of trace PAHs [15]. Li, Zhu, Chen, Ma, and Li (2018) synthesized a cyclodextrin-modified MOF (CD-MOF) as an SPE absorbent to extract sulfonamide (SA) from pork [16]. Despite the utilization of MOFs in food safety testing, challenges persist, such as complex procedures involving centrifugation and the challenging recovery from mixed solutions in practical applications [17].

Integrating MOFs with magnetic NPs (MNPs) offers several advantages for addressing these limitations. The utilization of an external magnet enhances the reproducibility of materials and facilitates particle separation from the reaction environment, eliminating the need for expensive, complex processes such as centrifugation and filtration [18,19,20]. To address these limitations, we developed a novel Fe_3_O_4_@MOF-545-AMSA nanomaterial designed for the efficient extraction and enrichment of highly carcinogenic HAAs, including 4,8-DiMeIQx, MeIQx, and IQ. Fe_3_O_4_@MOF-545-AMSA is synthesized through the interaction between the -NH_2_ group of aminomethanesulfonic acid (AMSA) and the acidic site of MOF-545, with the -SO_3_H group binding to the -NH_2_ group of the HAAs to enhance their enrichment in processed meat products.

In this study, we innovatively integrated the synthesized Fe_3_O_4_@MOF-545-AMSA with an automated workstation to construct a brand-new system for enriching HAAs in meat products. Compared with previously reported adsorbents (Table S1), Fe_3_O_4_@MOF-545-AMSA shows better overall adsorption performance. It requires a lower dosage (3 mg), and its equilibrium time (10 min) and desorption time (5 min) are shorter. This system breaks through traditional methods and automates the enrichment process, offering remarkable advantages such as improved detection efficiency, reduced human errors, and lower costs due to automation. Meanwhile, by combining MSPE with UPLC-MS/MS, we have developed a high-throughput detection method that can quickly and accurately identify multiple HAAs in meat products, providing new perspectives for food safety detection.

## 2. Results and Discussion

### 2.1. Fe_3_O_4_@MOF-545 and Fe_3_O_4_@MOF-545-AMSA Characterization

**Figure 1:**
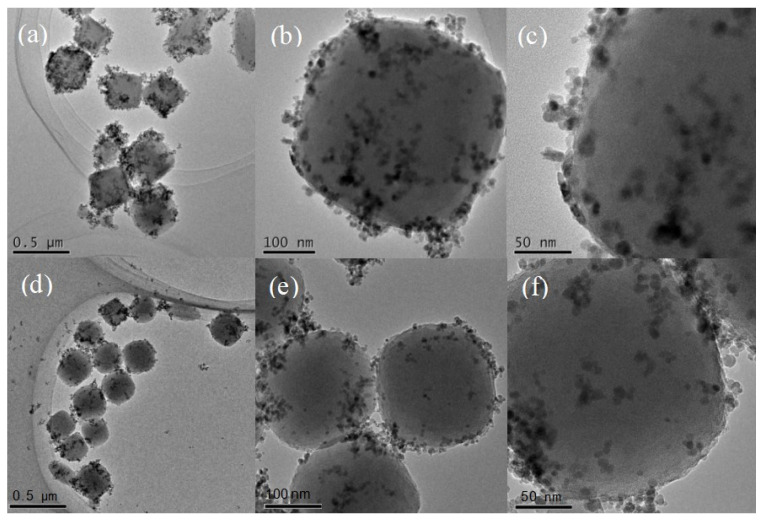
Transmission electron microscopy images of the synthesized (**a**–**c**) Fe_3_O_4_@MOF-545 and (**d**–**f**) Fe_3_O_4_@MOF-545-AMSA particles.

**Figure 2:**
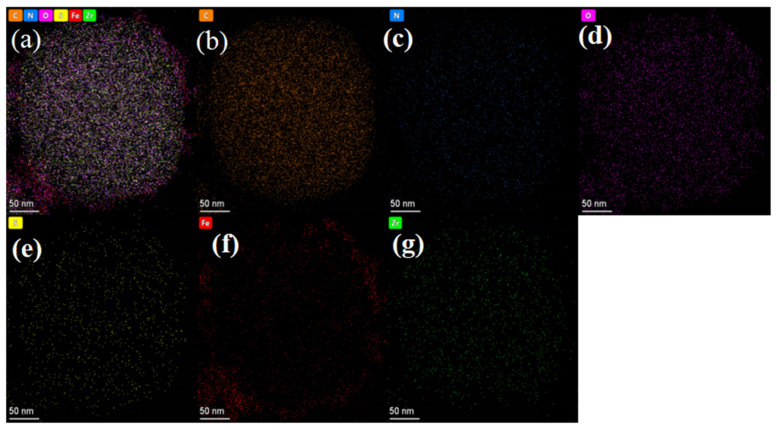
Elemental mapping of Fe_3_O_4_@MOF-545-AMSA: (**a**) combination diagram, (**b**) C, (**c**) N, (**d**) O, (**e**) S, (**f**) Fe, (**g**) Zr.

TEM was employed to analyze the morphological characteristics of Fe_3_O_4_@MOF-545 and Fe_3_O_4_@MOF-545-AMSA (Figure 1). Both samples displayed square-shaped nanoparticles of uniform size with consistent surface morphologies, indicating the encapsulation of magnetic Fe_3_O_4_ within MOF-545 shells. However, as can be seen from Figure 1a–c (Fe_3_O_4_@MOF-545) and Figure 1d–f (Fe_3_O_4_@MOF-545-AMSA), no significant difference is observed in AMSA attachment before and after attachment, as the particle sizes of both materials are in the range of 300–400 nm. Energy-dispersive X-ray spectroscopy (Figure 2) was employed to examine the Fe_3_O_4_@MOF-545-AMSA composite to determine the success of AMSA connection in Fe_3_O_4_@MOF-545. These findings confirmed the structural NP characteristics, including the compositional and geometrical distribution (Fe, S, Zr, O, N, and C), and AMSA was successfully linked to Fe_3_O_4_@MOF-545. Meanwhile, the scanning element content intensity mapping can also corroborate the successful synthesis of Fe_3_O_4_@MOF-545-AMSA (Figure S1).

**Figure 3:**
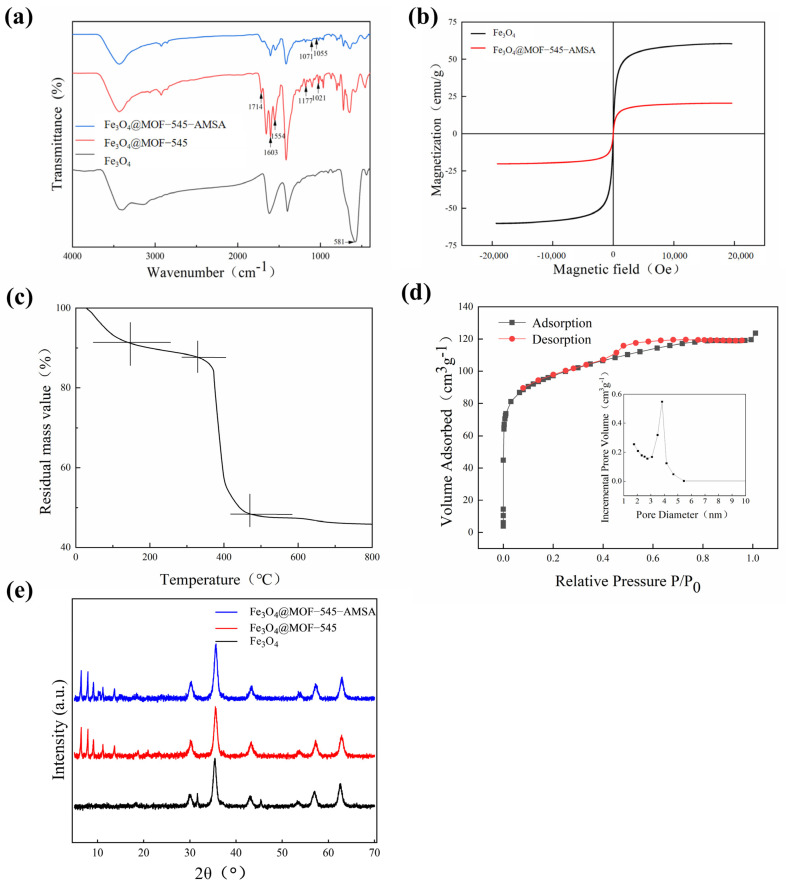
(**a**) Fourier-transform infrared (FTIR) spectra of Fe_3_O_4_, Fe_3_O_4_@MOF-545, and Fe_3_O_4_@MOF-545-AMSA. (**b**) Magnetization curves of Fe_3_O_4_ and Fe_3_O_4_@MOF-545-AMSA. (**c**) Thermogravimetric curves of Fe_3_O_4_@MOF-545-AMSA. (**d**) N_2_ adsorption−desorption isotherms of Fe_3_O_4_@MOF-545-AMSA (the inset shows the pore size distribution of Fe_3_O_4_@MOF-545-AMSA). (**e**) Widea-ngle XRD patterns of Fe_3_O_4_, Fe_3_O_4_@MOF-545, and Fe_3_O_4_@MOF-545-AMSA.

Figure 3a illustrates the FTIR spectra of Fe_3_O_4_, Fe_3_O_4_@MOF-545, and Fe_3_O_4_@MOF-545-AMSA, which offer insights into their chemical structures. The recorded spectra displayed a distinct absorption peak at 581 cm^−1^, signifying the stretching vibration of Fe-O-Fe bonds in the three materials [21]. Furthermore, in the spectrum of Fe_3_O_4_@MOF-545, new characteristic peaks were observed at 1021 cm^−1^, 1177 cm^−1^, 1603 cm^−1^, and 1714 cm^−1^. These peaks denote the vibrations of the benzene ring and the MOF porphyrin ring [22,23], respectively, demonstrating the presence of MOF-545. Additionally, the 1055 cm^−1^ and 1071 cm^−1^ peaks in the Fe_3_O_4_@MOF-545-AMSA spectrum were attributed to the skeletal vibrations of S-O [24], indicating AMSA’s bonding to Fe_3_O_4_@MOF-545. These characteristic peak phenomena in the FTIR spectra collectively demonstrate the successful synthesis of the Fe_3_O_4_@MOF-545 and Fe_3_O_4_@MOF-545-AMSA.

A VSM was used to assess the magnetic characteristics of Fe_3_O_4_ and Fe_3_O_4_@MOF-545-AMSA, while the magnetic field was used to plot the magnetization (Figure 3b). The hysteresis curve displayed no discernible post-hysteresis effects, suggesting that the material demonstrated excellent superparamagnetism. The saturation magnetization values were 60.47 emu/g for Fe_3_O_4_ and 20.53 emu/g for Fe_3_O_4_@MOF-545-AMSA. The TGA results reflected the mass ratios and thermal stability of the material components (Figure 3c). Three significant weight reductions were evident in Fe_3_O_4_@MOF-545-AMSA. The first, around 150 °C, was attributed to the physical desorption of water or solvent molecules [25,26], reducing the total mass by 8.9%. The second, around 330 °C, was related to the loss of oxygen-containing groups such as -OH [27], decreasing the total mass by 3.5%. The third, at approximately 470 °C, reduced the mass by 39.2%. During this stage, the material mass decreased rapidly, indicating metal–organic skeleton material decomposition and skeleton collapse [28].

The nitrogen adsorption isotherms (Figure 3d) were used to investigate the Fe_3_O_4_@MOF-545-AMSA pore structure and porosity, which yielded a 361 m^2^/g BET surface area, a 2.05 nm average pore size, and a 0.18 cm^3^ g^−1^ pore volume. In comparison to the BET surface area (120 m^2^/g) of Fe_3_O_4_@MOF-545 from our prior study, the BET surface area of Fe_3_O_4_@MOF-545-AMSA is optimized to 361 m^2^/g, suggesting more favorable conditions for molecular adsorption [29]. The material exhibited significant surface area and porosity, facilitating the formation of several routes for HAA adsorption. These findings indicated that Fe_3_O_4_@MOF-545-AMSA was suitable for effective HAA adsorption.

The synthesized crystal structures and phase purities were further investigated by characterizing the X-ray single-crystal diffraction (XRD) of Fe_3_O_4_, Fe_3_O_4_@MOF-545, and Fe_3_O_4_@MOF-545-AMSA (Figure 3e). The peaks at 2 θ =30.1°, 35.4°, 43.1°, 53.4°, 57.0°, and 62.7° can be assigned to the (220), (311), (400), (422), (511), and (440) characteristic diffraction peaks of the Fe_3_O_4_ nanoparticles, respectively (JCPDS no. 19-629). A comparison of XRD analysis of Fe_3_O_4_@MOF-545 revealed that no impurity peaks were found, except for the crystalline Fe_3_O_4_ and MOF-545 peaks (at 6.2°, 7.9°, 9.1°, 10.9° and 13.6°), which indicated that the two substances demonstrated a high degree of direct crystallinity, and the MOF-545 was perfectly integrated with the host material. Moreover, no significant discrepancy was detected between Fe_3_O_4_@MOF-545-AMSA and Fe_3_O_4_@MOF-545. This further confirms that the introduction of AMSA did not cause damage to the crystal structure of Fe_3_O_4_@MOF-545.

Based on the above characterization results, the adsorption mechanism of HAAs was systematically explained. As shown in Table S2, the three HAAs have similar molecular structures, all with aromatic rings. Fe_3_O_4_@MOF-545-AMSA has a porphyrin ring, and there is a π–π conjugation between the aromatic rings of the HAAs and this porphyrin ring [29]. Figure S2 showed that Fe_3_O_4_@MOF-545-AMSA was effective in absorbing three HAAs. However, the three HAAs were not obviously adsorbed on Fe_3_O_4_@PVP, indicating π–π conjugation and the synergistic effect of pore structure played an important role in HAAs separation (Figure 3d).

### 2.2. Optimization of the MSPE Conditions

**Figure 4:**
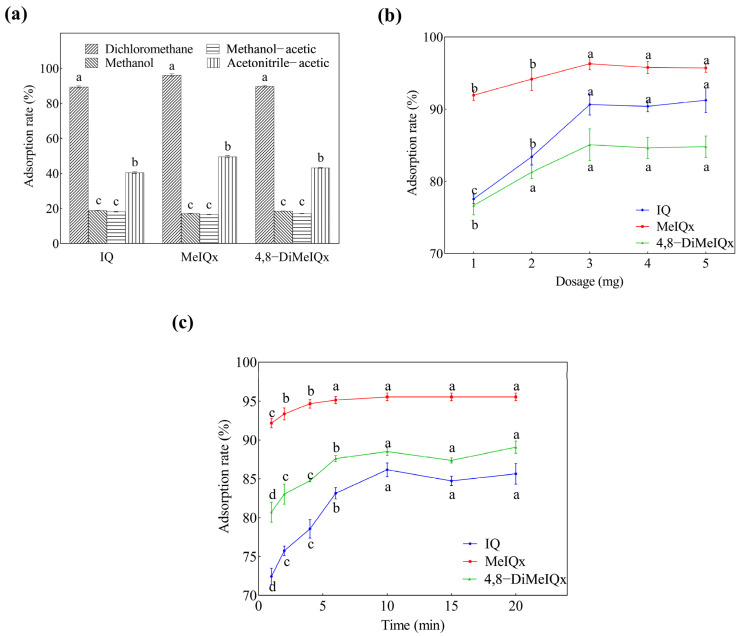
(**a**) Effect of the variety of extracting agents, (**b**) adsorbent amount, and (**c**) extraction time on IQ, MeIQx, and 4,8-DiMeIQx adsorption from Fe_3_O_4_@MOF-545-AMSA. Different small letters indicate significant difference (*p* < 0.05).

The study investigated the influence of different extraction agents, adsorbent quantities, and extraction durations on the adsorption rates of 4,8-DiMeIQx, IQ, and MeIQx to determine the optimal conditions for extracting HAAs (Figure 4). The selection of the extraction agent notably impacted the adsorption capacity of the substance [30]. Four organic solvents, including methanol, dichloromethane, 9:1 *v*/*v* acetonitrile–acetic acid and 9:1 *v*/*v* methanol–acetic acid, were used as HAA extractants. As shown in Figure 4a, dichloromethane significantly enhanced the adsorption rates compared to the other extractants, yielding values of 89.9, 89.8% and 96.2% for 4,8-DiMeIQx, IQ and MeIQx, respectively. Therefore, it was inferred that a polar force existed between the material and HAAs. The Fe_3_O_4_@MOF-545-AMSA quantity significantly influenced the adsorption cost [29]. A higher HAA separation efficacy was evident, as the Fe_3_O_4_@MOF-545-AMSA weight increased from 1 mg to 5 mg. At 1, 2, 3, 4 and 5 mg, Fe_3_O_4_@MOF-545-AMSA displayed adsorption efficiency values of 76.2, 82.3, 89.8, 89.7 and 90.0% for IQ, 91.2, 92.4, 96.2, 95.8 and 96.3% for MeIQx, and 75.4, 80.6, 83.2, 83.3 and 83.5% for 4,8-DiMeIQx, respectively (Figure 4b). The optimal dosage of Fe_3_O_4_@MOF-545-AMSA was determined to be 3 mg, as there was no significant increase in the adsorption rate beyond this amount. Additionally, the adsorption rates of the adsorbents were influenced by the adsorption time, with the Fe_3_O_4_@MOF-545-AMSA adsorption rate on the HAAs showing a gradual increase with prolonged adsorption time. The Fe_3_O_4_@MOF-545-AMSA adsorption sites were occupied by IQ after 10 min, MeIQx after 6 min, and 4,8-DiMeIQx after 10 min, respectively (Figure 4c). Therefore, an adsorption duration of 10 min was selected as optimal.

**Figure 5:**
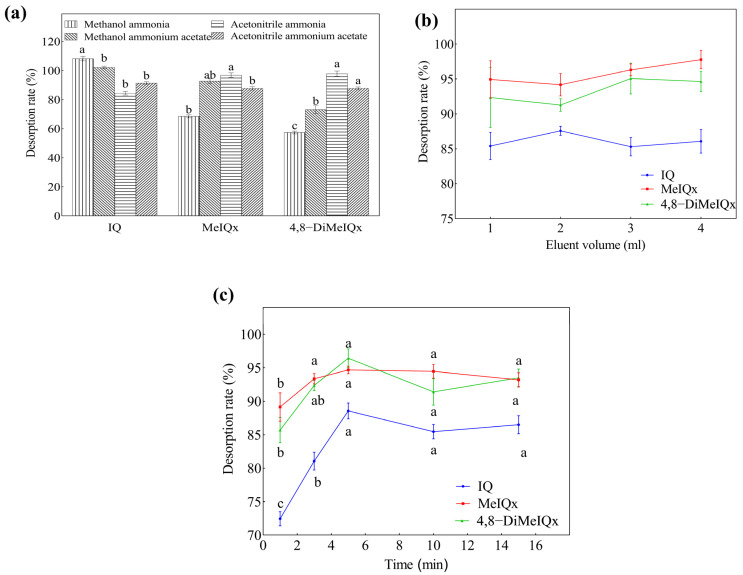
Effect of (**a**) the variety of desorption agents, (**b**) volume of the desorption agent, and (**c**) desorption time on IQ, MeIQx, and 4,8-DiMeIQx desorption from Fe_3_O_4_@MOF-545-AMSA. Different small letters indicate significant difference (*p* < 0.05).

The impact of various desorption agents and their respective volume, as well as the desorption time, on the IQ, MeIQx, and 4,8-DiMeIQx desorption rates was examined to determine the optimal desorption conditions (Figure 5). Some studies suggest that enhancing the desorption rate of HAAs can be achieved by incorporating a basic solution. Thus, in this study, NH_3_·H_2_O and ammonium acetate were introduced as desorption agents in methanol and acetonitrile, with the ratio of the four desorption agents set at 9:1 *v*/*v* organic phase–inorganic phase. The results (Figure 5a) indicated that the acetonitrile-NH_3_·H_2_O combination showed the most effective desorption ability among the three HAAs. As shown in Figure 5b, higher desorption agent volumes did not significantly increase the desorption rates of the three HAAs. For economic and environmental protection, a 1 mL desorption agent was selected for desorption. As shown in Figure 5c, more than 88% target analyte elution occurred within 5 min, indicating that Fe_3_O_4_@MOF-545-AMSA could obtain desorption equilibrium in a short time frame.

### 2.3. Selectivity, Reproducibility, and Stability of Fe_3_O_4_@MOF-545-AMSA

**Figure 6:**
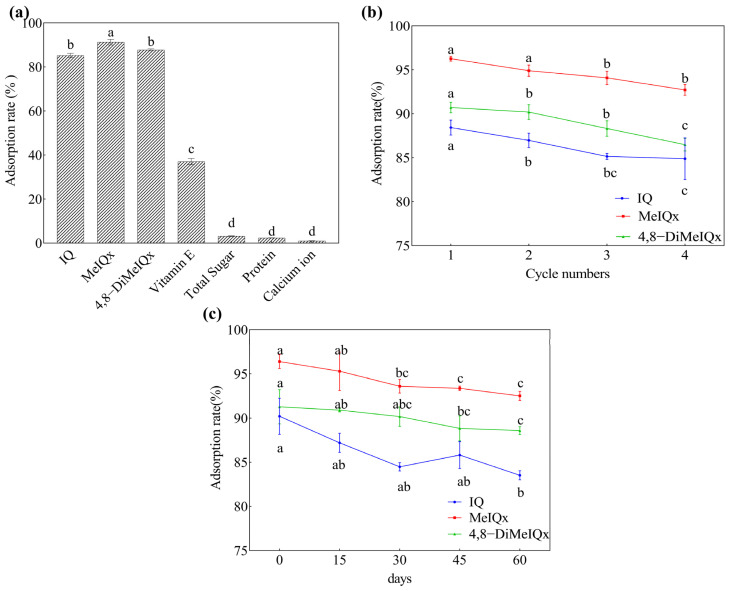
Effect of (**a**) interferents, (**b**) cycle numbers, and (**c**) storage time on IQ, MeIQx, and 4,8-DiMeIQx adsorption from Fe_3_O_4_@MOF-545-AMSA. Different small letters indicate significant difference (*p* < 0.05).

Given the intricate nature of the food matrix, the meticulous selection of materials is crucial for assessing their efficacy [31]. Vitamin E, sugar, protein, and calcium ions, which may coexist with HAAs in meat products, were chosen as potential interferents to mimic the meat matrix. The 4,8-DiMeIQx, IQ, and MeIQx adsorption rates were significantly higher than the interferents (Figure 6a), indicating that the presence of impurities in meat products did not affect the efficacy of Fe_3_O_4_@MOF-545-AMSA, suggesting its high selectivity. Fe_3_O_4_@MOF-545-AMSA rarely adsorbs large, non-aromatic, non-basic compounds. Good reproducibility and stability are vital characteristics of new materials [32,33]. The reproducibility of Fe_3_O_4_@MOF-545-AMSA was assessed through multiple experiments involving acetone rinsing before each cycle. As shown in Figure 6b, Fe_3_O_4_@MOF-545-AMSA maintains an adsorption rate of over 80% for the three HAAs after four cycles, indicating good reproducibility. The Fe_3_O_4_@MOF-545-AMSA was stored for two months to assess its stability. Sampling occurred at 15-day intervals to evaluate the HAA extraction efficiency. The material consistently maintained an extraction efficiency of over 80%, even with prolonged storage, demonstrating its exceptional stability (Figure 6).

### 2.4. Method Validation

Based on the concentration of HAAs in the actual sample, the HAA recovery rates were acquired using Dezhou braised chicken, spiced beef, fried pork, and crispy yellow croaker samples spiked with 10 μg/kg (low), 25 μg/kg (medium), and 50 μg/kg (high) standard solution concentrations (Table 1). The results showed HAA recovery rates of between 83.7% and 111.0%, indicating that the method was highly accurate [1]. The relative standard deviations (RSDs) of all samples were below 6%, indicating the high precision of the method. In conclusion, this method is accurate, reproducible, and suitable for determining HAAs in different meat products. The LODs of the HAAs ranged from 0.1 μg/kg to 0.5 μg/kg, and the LOQs ranged from 0.3 μg/kg to 1.0 μg/kg (Table S3).

**Figure 7:**
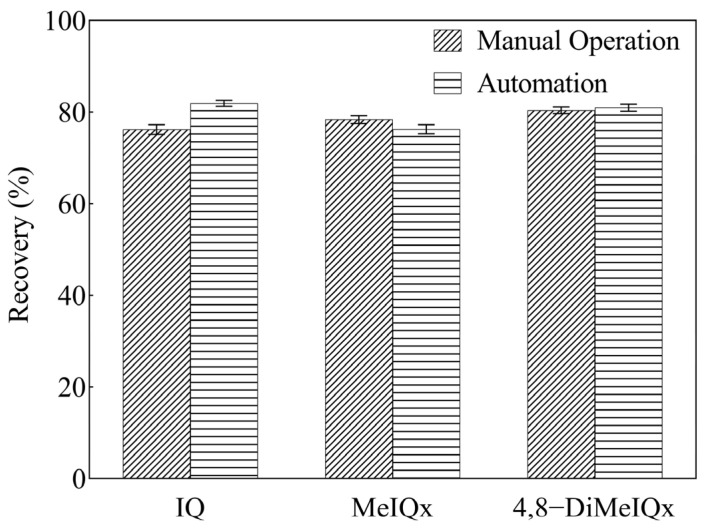
Comparison of recovery rates of three heterocyclic aromatic amines via automated extraction and manual operation.

The feasibility of the high-throughput automated extraction process was confirmed by implementing the optimal extraction conditions on an automated extraction instrument and comparing the extraction results of HAAs between the automated and manual methods. Firstly, the standards were validated. As shown in Figure 7, automated extraction had minimal effects on the extraction efficiency of the three HAA standards, with recovery rates relatively close to those of manual extraction. Table S4 shows the recovery rates and RSDs of the three HAAs extracted from IQ, MeIQx, and 4,8-DiMeIQx using a fully automated extractor. The recovery rates ranged from 75.2% to 87.8%, while the RSDs were lower than 2%, indicating that the extraction process is highly reproducible.

The method was utilized to extract HAAs from four different meat samples to verify its efficacy in automated extraction with real samples. Subsequently, the results were compared with those obtained manually for three HAAs. Comparable results were observed between the two extraction methods across various levels of 4,8-DiMeIQx, IQ, and MeIQx (Table 1, Table 2 and Table S5). The content of HAAs closely matched the manually measured values, with relative standard deviations (RSDs) below 5%, demonstrating the high accuracy of this extraction process for detecting HAAs in real samples.

### 2.5. Comparison Between Fe_3_O_4_@MOF-545-AMSA and SPE

To assess the extraction efficiency of materials against established application standards, a comparison was made between the conventional solid-phase extraction column method, the novel dispersion solid-phase extraction method, and Fe_3_O_4_@MOF-545-AMSA for extracting HAAs. The results are summarized in Table 3. Overall, the HAA recovery rates using the three enrichment methods follow this order: dispersion solid-phase extraction > magnetic materials > traditional solid-phase extraction, with consistently reproducible outcomes. The magnetic material demonstrates comparable performance to the dispersion solid-phase extraction method, which is considered superior in the current market. Notably, the recovery rate of the magnetic material for 4,8-DiMeIQx surpasses that of the dispersion solid-phase extraction method. Moreover, the magnetic material simplifies complex pretreatment steps such as centrifugation, is cost-effective, and lends itself well to automation. In contrast, the manual flow rate control in the traditional solid-phase extraction method using a solid-phase extraction column often leads to the incomplete adsorption of HAA solutions, resulting in lower recovery rates. In conclusion, the magnetic material shows promise for HAA extraction applications.

## 3. Experimental Section

### 3.1. Materials and Reagents

Ferric (III) chloride hexahydrate, ferric (II) chloride tetrahydrate, 99.9% zirconyl chloride octahydrate, 98% ammonium acetate, ammonia (NH_3_·H_2_O), trisodium citrate dihydrate, 99.5% benzoic acid, and 99.5% nitrogen–nitrogen dimethylformamide (DMF) were provided by (Fuchen Reagent, Tianjin, China). PVP (Mw = 40,000), 98% AMSA, and 97% tetrakis(4-carboxyphenyl)porphyrin (TCPP) were provided by (Macklin, Shanghai, China). All reagents were analytically pure. Chromatographic-grade acetic acid, acetonitrile, methanol, dichloromethane, and ethanol were provided by (Mreda, Beijing, China), while IQ, MeIQx, and 4,8-DiMeIQx were purchased from TRC (Downsview, ON, Canada). Table S2 shows the chemical names, abbreviated names, and structures of the three selected HAAs. Dezhou braised chicken, spiced beef, fried pork, and crispy yellow croaker were obtained from a supermarket in Beijing, China.

### 3.2. Equipment

An FEI TF20 instrument (Hillsboro, OR, USA) was used for transmission electron microscopy (TEM). Fourier-transform infrared spectroscopy (FTIR) was conducted using an FTIR spectrometer (Perkin Elmer Frontier, Waltham, MA, USA), with spectra collected at 25 °C in a 400–4000 cm^−1^ range. A vibrating sample magnetometer (VSM, LakeShore 7404, OH, USA) was employed for magnetic hysteresis loop analysis. A Rigaku TG-DTA8122 thermal gravimetric analyzer (Tokyo, Japan) was employed for thermogravimetric analysis (TGA) over a 30–800 °C range under air conditions at a rate of 10 °C/min. The sample pore structures were assessed (Micromeritics ASAP 2020 HD88 adsorption system, GA, USA) to obtain the N_2_ adsorption–desorption isotherms, while the specific surface area was determined via the Brunauer–Emmett–Teller (BET) method. A Rigaku Ultima IV X-ray diffractometer (Tokyo, Japan) was used for X-ray diffraction (XRD) to analyze the crystal structures, identify the phase compositions, and evaluate the crystallinity of the samples. This method combined a highly efficient Waters Acquity I-Class liquid chromatograph with an exceptionally sensitive Xevo TQ-S tandem mass spectrometer, using a 2.5 μm XSELECT CSH XP column (Milford, Massachusetts, USA) for separation. A Purifier HT instrument (Genfine, China) was used to extract and purify the nucleic acids for high-throughput automated HAA enrichment. Table S6 outlines the high-throughput automated extraction program.

### 3.3. Synthesis of Fe_3_O_4_@MOF-545-AMSA

**Scheme 1:**
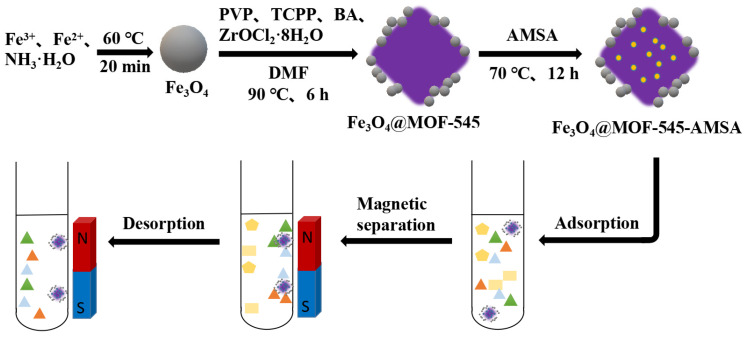
The Fe_3_O_4_@MOF-545-AMSA synthesis scheme and the enrichment processes.

Fe_3_O_4_@MOF-545-AMSA was synthesized using a previously described chemical co-precipitation method with slight modifications [24,29]. First, 32.6 g FeCl_2_·4H_2_O and 53.17 g FeCl_3_·6H_2_O were dissolved in 1.5 L deionized water and 2 L ethanol, after which 200 mL of ammonium hydroxide was added at 60 °C for 20 min, and then the Fe_3_O_4_ NPs were washed with a 1% sodium citrate solution, deionized water, and ethanol, respectively. Next, 6 mg mL^−1^ of Fe_3_O_4_ NPs, 30 mg mL^−1^ of PVP, 30 mg mL^−1^ of TCPP, 15 mg mL^−1^ of ZrOCl_2_·8H_2_O, and 280 mg mL^−1^ of benzoic acid were mixed for 6 h at 90 °C to obtain Fe_3_O_4_@MOF-545. The Fe_3_O_4_@MOF-545 was washed three times with DMF, followed by dissolution in 40 mL ethanol, after which 0.1 g AMSA was added. The mixture was refluxed for 12 h at 70 °C while stirring constantly, after which the Fe_3_O_4_@MOF-545-AMSA product was rinsed once using deionized water and twice with ethanol. Figure S2 shows the adsorption of HAAs using the Fe_3_O_4_, Fe_3_O_4_@MOF-545, and Fe_3_O_4_@MOF-545-AMSA adsorbents. Compared with other MOFs used for the adsorption of HAAs, the synthesis process of Fe_3_O_4_@MOF-545-AMSA has advantages such as time-saving and mild preparation conditions [1]. The synthesis and adsorption procedures are shown in Scheme 1.

### 3.4. IQ, MeIQx and 4,8-DiMeIQx Separation Fe_3_O_4_@MOF-545-AMSA

The Fe_3_O_4_@MOF-545-AMSA adsorbent was employed for rapid 4,8-DiMeIQx, IQ, and MeIQx extraction. Here, 3 mg of Fe_3_O_4_@MOF-545-AMSA was mixed with a 2 mL IQ, MeIQx, and 4,8-DiMeIQx solution (50.0 μg L^−1^) to examine the extraction and elution agent variety, adsorbent quantity, reaction time, and elution volume. Scheme 1 illustrates the synthesis and enrichment processes. The Fe_3_O_4_@MOF-545-AMSA adsorption and desorption rates were calculated using Equations (1) and (2):

(1) $$R_{1}(\%)=\frac{C_{0}-C_{t}}{C_{0}}\times 100$$

(2) $$R_{2}(\%)=\frac{C_{e}}{C_{0}-C_{t}}\times 100$$

where *C*_0_ (μg L^−1^) denotes the initial concentration, *C_t_* (μg L^−1^) represents the residual concentration after adsorption, and *C*_e_ (μg L^−1^) denotes the post-desorption concentration.

### 3.5. MSPE Pretreatment

Dezhou braised chicken, spiced beef, fried pork, and crispy yellow croaker were selected as food samples for the separation analysis. A 2 g meat sample was dissolved in a 20 mL solution of dichloromethane and 1 mol L^−1^ sodium hydroxide (4:1, *v*/*v*). The solution was homogenized and vortexed, and the supernatant was collected via centrifugation. Subsequently, a 10 mL sample was mixed with 3 mg of Fe_3_O_4_@MOF-545-AMSA for 10 min. The Fe_3_O_4_@MOF-545-AMSA was then separated from the mixture using an external magnet and transferred to a solution of 1 mL acetonitrile and NH_3_·H_2_O (9:1, *v*/*v*) for desorption of the three HAAs. Isolated HAAs were then detected using UPLC-MS/MS.

### 3.6. Analysis Using UPLC-MS/MS

The mobile phases consisted of a mixture of 0.03 mol L^−1^ acetic acid and ammonium acetate (pH 5.0) (A) and acetonitrile (B) in gradient elution mode at a 0.3 mL min^−1^ flow rate, a 40 °C column temperature, and a 5 μL injection volume. Table S7 presents the gradient and ESI-MS conditions. Standard solutions of 4,8-DiMeIQx, IQ, and MeIQx were prepared at different concentrations. The UPLC-MS/MS results are shown in Figure S3. Each sample was analyzed in triplicate using UPLC-MS/MS to determine the method’s limit of detection (LOD), linearity, limit of quantification (LOQ), and standard deviation.

### 3.7. Statistical Analysis

All experiments were repeated three times, and the data were expressed as mean ± standard deviation. The variation among the samples was statistically assessed via one-way analysis of variance (ANOVA) and Student’s *t*-test (*t*-test) using SPSS 17.0 (Chicago, IL, USA).

## 4. Conclusions

This study involved the synthesis of a novel magnetic Fe_3_O_4_@MOF-545-AMSA for the enrichment of three HAAs (4,8-DiMeIQx, MeIQx, and IQ) in thermally processed meat products, followed by UPLC-MS/MS analysis. The study identified optimal extraction conditions utilizing dichloromethane as the extraction agent, 3 mg of adsorbent, and a 10 min extraction duration. The ideal desorption conditions involved acetonitrile–NH_3_·H_2_O as the desorption agent at a volume of 1 mL and a desorption time of 5 min. The nanomaterial exhibited a high specific surface area, excellent chemical stability, superparamagnetism, and other characteristics suitable for automated extraction equipment. Comparative and validation experiments verified the effectiveness of this magnetic material in extracting and detecting HAAs in actual samples. Thus, the developed automated high-throughput extraction process offers a promising and efficient approach for monitoring HAAs in processed meats. Building on these current achievements, we plan to further optimize the process parameters and explore the application of this technology in more complex food systems. This is likely to introduce innovative solutions for food safety detection and steadily raise the safety standards of the food industry.

## Figures and Tables

**Table 1:** Analytical results of IQ, MeIQx, and 4,8-DiMeIQx in four different meat products (n = 6).

Sample	Analytes	Found (ng/g)	Spiked Level (μg/kg)					
			10		25		50	
			Recovery (%)	RSD (%)	Recovery (%)	RSD (%)	Recovery (%)	RSD (%)
Dezhou braised chicken	IQ	0.98	93.40	1.48	92.80	3.67	90.00	5.49
	MeIQx	N.D.	91.70	1.03	88.70	4.35	93.20	2.72
	4,8-DiMeIQx	N.D.	85.50	4.29	85.30	2.77	84.60	2.87
Spiced beef	IQ	1.50	89.70	1.58	93.00	2.17	90.60	2.56
	MeIQx	N.D.	99.30	2.13	93.40	1.44	99.10	3.87
	4,8-DiMeIQx	N.D.	83.70	1.43	88.40	4.81	86.80	3.03
Fried pork	IQ	1.66	93.70	3.04	98.60	2.11	87.90	3.21
	MeIQx	0.58	103.80	1.49	95.10	3.07	111.00	3.85
	4,8-DiMeIQx	N.D.	89.60	4.76	93.50	3.32	98.90	3.25
Crispy yellow croaker	IQ	2.40	91.10	4.21	97.10	2.70	96.40	3.36
	MeIQx	N.D.	92.20	1.87	84.80	4.24	107.30	4.26
	4,8-DiMeIQx	0.44	87.30	2.41	88.50	4.33	89.40	3.08

N.D.: not detected.

**Table 2:** Automated extractor detection of heterocyclic aromatic amines in four different meat products (n = 3).

Sample Name	Dezhou Braised Chicken	Spiced Beef	Fried Pork	Crispy Yellow Croaker
IQ (ng/g)	1.142	1.463	1.591	2.448
MeIQx (ng/g)	N.D.	N.D.	0.551	N.D.
4,8-DiMeIQx (ng/g)	N.D.	N.D.	N.D.	0.482

N.D.: not detected.

**Table 3:** Comparison of recovery and precision of three enrichment methods on three heterocyclic aromatic amines (%), (n = 6).

HAAs	Fe_3_O_4_@MOF-545-AMSA		Bond Elut PPL		QuEChERS	
	Recovery	RSD	Recovery	RSD	Recovery	RSD
IQ	78.77	1.44	48.87	1.50	81.47	1.47
MeIQx	81.35	2.05	39.09	1.80	87.96	0.76
4,8-DiMeIQx	80.16	1.39	57.47	1.93	79.42	1.53

## Data Availability

The original contributions presented in the study are included in the article. Further inquiries can be directed to the corresponding author.

## Supplementary Materials

The following supporting information can be downloaded at https://www.mdpi.com/article/10.3390/molecules30081705/s1, Figure S1: Scanning element content intensity map; Figure S2: Adsorption of heterocyclic aromatic amines with three different adsorbents; Figure S3: Multiple reaction monitoring (MRM) chromatograms of three types of HAAs; Table S1: HAAs adsorption capacities of Fe_3_O_4_@MOF-545-AMSA [1,34,35,36]; Table S2: Chemical name, abbreviated name, and structure of the three selected heterocyclic aromatic amines (HAAs); Table S3: The LOD and LOQ of heterocyclic aromatic amines in four different meat products (µg/kg), (n = 3); Table S4: Automated verification of the recovery rate and relative standard deviation of heterocyclic aromatic amines (HAAs) standards; Table S5: Automated extraction instrument for detecting the content and relative standard deviation of heterocyclic aromatic amines (HAAs) in four different meat products (n = 8); Table S6: Table of high-throughput automated extraction program; Table S7: Overview of the gradient condition parameters (a) and ESI-MS condition parameters (b).
