# Supplementary material for: Highly Efficient Enrichment of Heterocyclic Aromatic Amines in Meat Products Using the Magnetic Metal—Organic Framework Fe3O4@MOF-545-AMSA
Source: Molecules. 2025 Apr 10;30(8):1705. doi: 10.3390/molecules30081705 (PMC12029920; doi:10.3390/molecules30081705)
Supplement: Supplementary file 1 [file molecules-30-01705-s001.zip › molecules-3546345-supplementary.pdf]

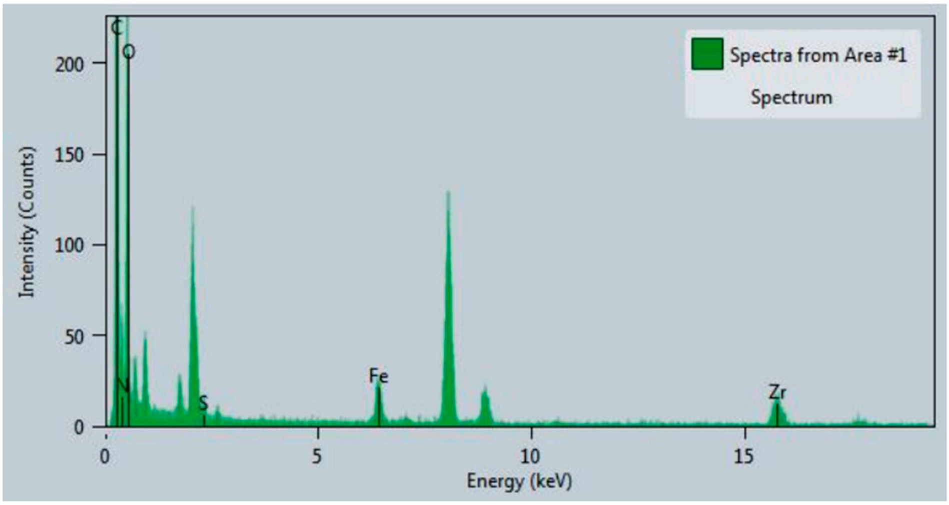

Figure S1 Scanning element content intensity map

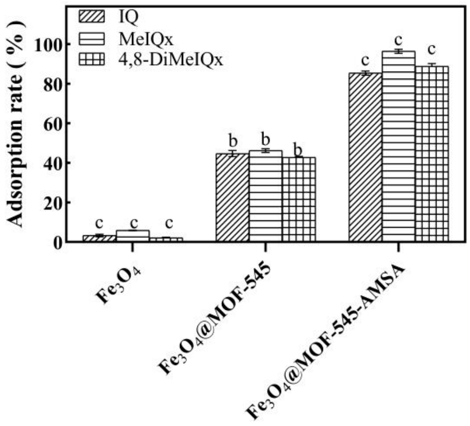

Figure S2. Adsorption of heterocyclic aromatic amines with three different adsorbents.Different small letters indicate significant difference (p<0.05).

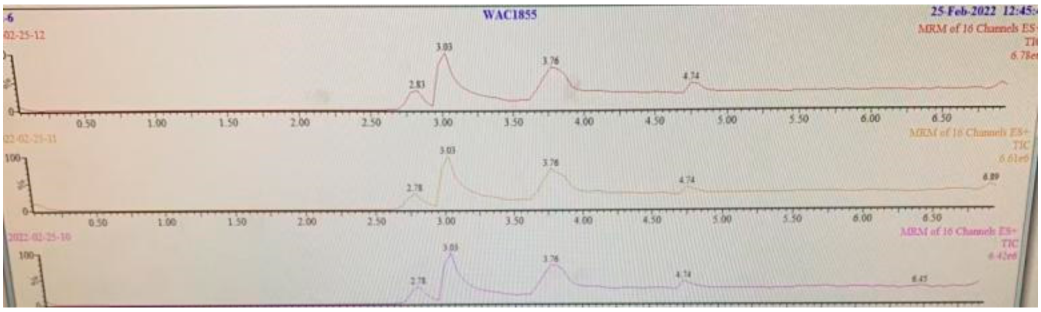

Figure S3 Multiple reaction monitoring (MRM) chromatograms of three types of HAAs.

Table S1 HAAs adsorption capacities of Fe_3O_4@MOF-545-AMSA.

nanomaterials	HAAs	Adsorbent amount (mg)	Equilibr-ium time (min)	Desorpti -on time	Recovery (%)	Ref
AG$^{D-UiO-66}$	12HAAs	10	30	10	94.65-107.56	[1]
Fe_3O_4@COF@Cys	5HAAs	10	30	30	90.4-102.8	[2]
Fe_3O_4@COF-COOH	16HAAs	4	20	9	62.12-126.86	[3]
Fe_3O_4@COP	6HAAs	10	5	20	82.0 – 109.5	[4]
Fe_3O_4@MOF-545-AMS-A	3HAAs	3	10	5	83.7–111.0	This study

Table S2. Chemical name, abbreviated name, and structure of the three selected heterocyclic aromatic amines (HAAs).

Chemical name	Abbreviated name	Structure
2-amino-3-methylimidazole [4,5-f]quinoline	IQ	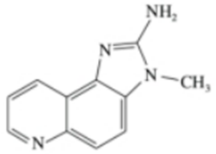
2-amino-3,8-dimethylimidazo[4,5-f]quinoline	MeIQx	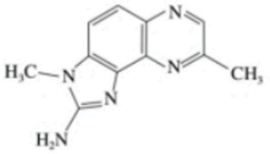
2-amino-3,4,8-dimethylimidazole-[4,5-f]-quinoxaline	4,8-DiMelQx	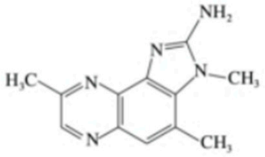

Table S3 The LOD and LOQ of heterocyclic aromatic amines in four different meat products (μg/kg), n=3.

**Sample name**	IQ		MeIQx		4,8-DiMeIQx	
	LOD	LOQ	LOD	LOQ	LOD	LOQ
Dezhou braised chicken	0.2	0.5	0.5	1.0	0.1	0.5
Spiced beef	0.1	0.5	0.3	1.0	0.5	1.0
Fried pork	0.2	0.5	0.1	0.3	0.5	1.0
Crispy yellow croaker	0.2	0.5	0.1	0.5	0.1	0.3

Table S4 Automated verification of the recovery rate and relative standard deviation of heterocyclic aromatic amines (HAAs) standards, (n=3).

HAAs	Recovery (%)	Mean value (%)	RSD (%)
IQ	75.2	79.5	1.02
	82.5		
	82.1		
	78.5		
	75.9		
	79.0		
	82.2		
	80.9		
MeIQx	79.9	79.8	1.53
	87.8		
	75.6		
	80.0		
	75.2		
	76.9		
	84.2		
	78.7		
4,8-DiMeIQx	82.1	80.5	1.18
	78.7		
	81.5		
	80.0		
	77.6		
	82.8		
	75.4		
	86.1		

Table S5 Automated extraction instrument for detecting the content and relative standard deviation of heterocyclic aromatic amines (HAAs) in four different meat products (n=8).

Sample	HAAs	Content (ng/g)	Mean value (ng/g)	RSD (%)
Dezhou braised chicken	IQ	1.164	1.142	3.84
		1.165		
		1.095		
		1.022		
		0.968		
		1.191		
		1.291		
		1.242		
	MeIQx	N.D.	N.D.	N.D.
	4,8-DiMeIQx	N.D.	N.D.	N.D.
Spiced beef	IQ	1.420	1.463	4.46
		1.478		
		1.492		
		1.620		
		1.632		
		1.335		
		1.464		
		1.265		
	MeIQx	N.D.	N.D.	N.D.
	4,8-DiMeIQx	N.D.	N.D.	N.D.
Fried pork	IQ	1.643	1.591	3.7
		1.509		
		1.620		
		1.755		
		1.630		
		1.401		
		1.554		
		1.615		
	MeIQx	0.550	0.551	2.35
		0.556		
		0.553		
		0.572		
		0.651		
		0.610		
		0.457		
		0.462		

	4,8-DiMeIQx	N.D.	N.D.	N.D.
Crispy yellow croaker	IQ	2.546<br>2.384<br>2.488<br>2.509<br>2.267<br>2.285<br>2.516<br>2.588	2.448	4.28
	MeIQx	N.D.	N.D.	N.D.
	4,8-DiMeIQx	0.479<br>0.468<br>0.500<br>0.546<br>0.437<br>0.411<br>0.524<br>0.490	0.482	1.55

N.D.:Not detected

Table S6 The table of high-throughput automated extraction program.

Step	Name	Plate	Mixing time (s)	Volume (μL)	Magnetic attraction time (s)	Waiting time (min)	Mixing speed
1	take magnetic beads	1	5	500	60	0	slow
2	adsorption	2	600	1000	60	0	slow
3	desorption	4	300	1000	60	0	slow
4	discard	5	0	500	0	0	slow

Table S7 Overview of the gradient condition parameters (a) and ESI-MS condition parameters (b)

(a) The gradient condition parameters of UPLC-MS/MS analysis

Program of Gradient Elution		
**Time (min)**	**Mobile phase A (%)**	**Mobile phase B (%)**
0	95.0	5.0
0.5	95.0	5.0
3.0	70.0	30.0
6.0	40.0	60.0
6.1	5.0	95.0
6.5	5.0	95.0
6.6	95.0	5.0
7.0	95.0	5.0

(b) The ESI-MS condition parameters of UPLC-MS/MS analysis

Basic parameters of mass spectrometer	
Ion mode	ESI, negative
Gas temperature	350 ℃
Scan type	multiple reaction monitoring (MRM)

MRM parameter table				
**RM**	**Precursor ion (m/z)**	**Product ion (m/z)**	**The cone hole voltage (V)**	**Collision voltage(CE)**
IQ	199.1	184.0[a]	40	35
		157.0	40	35
MeIQx	213.9	130.9[a]	40	35
		199.0	40	35
4,8-DiMeIQx	227.9	159.8[a]	40	35
		212.1	40	35

[a] represents quantitative ion

References

[1] Zhao, Q.; Hou, M.H.; Zhang, L.G.; et al. Defective UiO-66/Cellulose Nanocomposite Aerogel for the Adsorption of Heterocyclic Aromatic Amines. Food Chemistry. **2024**, 449, 139225.

[2] Fan, Y.M.; Shi, Y.H.; He, R.; et al. Simultaneous Detection of Heterocyclic Aromatic Amines and Acrylamide in Thermally Processed Foods by Magnetic Solid-Phase Extraction Combined with HPLC-MS/MS Based on Cysteine-Functionalized Covalent Organic Frameworks. Food Chemistry. **2023**, 424, 136349.

[3] Wang, T.; Liu, W.; Chen, L.; et al. A Magnetic Carboxyl-Functionalized Covalent Organic Framework for the Efficient Enrichment of Foodborne Heterocyclic Aromatic Amines prior to UPLC-MS Analysis. Food Chemistry. **2024**, 461, 140852.

[4] Feng, Y.,Xu, Y.; Li, W.; Chen, S.; Su, Z.; Xi, L.; Li, G. Improved enrichment and analysis of heterocyclic aromatic amines in thermally processed Foods by magnetic solid phase extraction combined with HPLC-MS/MS. Food Control. **2022**, 137, 108929.
